# Unusual pattern of chikungunya virus epidemic in the Americas, the Panamanian experience

**DOI:** 10.1371/journal.pntd.0005338

**Published:** 2017-02-21

**Authors:** Jean-Paul Carrera, Yamilka Díaz, Bernardino Denis, Itza Barahona de Mosca, Dennys Rodriguez, Israel Cedeño, Dimelza Arauz, Publio González, Lizbeth Cerezo, Lourdes Moreno, Lourdes García, Lisseth E. Sáenz, María Aneth Atencio, Eddy Rojas-Fermin, Fernando Vizcaino, Nicolas Perez, Brechla Moreno, Sandra López-Vergès, Anayansi Valderrama, Blas Armién

**Affiliations:** 1 Department of Research in Virology and Biotechnology, Gorgas Memorial Institute of Health Studies; Panama City, Panama; 2 Department of Pre-clinical Sciences, School of Medicine, Columbus University; Panama City, Panama; 3 Department of Research in Emerging and Zoonotic Diseases, Gorgas Memorial Institute of Health Studies; Panama City, Panama; 4 General Direction of the Ministry of Health; Panama City, Panama; 5 National Department of Epidemiology, Ministry of Health; Panama City, Panama; 6 Immunovirology section, Public Health Reference Laboratory, Gorgas Memorial Institute of Health Studies; Panama City, Panama; 7 Vector-Control National Department, Ministry of Health; Panama City, Panama; 8 Health Center Puerto Obaldía; Panama City, Panama; 9 Department of Research in Medical Entomology, Gorgas Memorial Institute of Health Studies; Panama City, Panama; 10 Research Direction, Universidad Interamericana de Panama; Panama City, Panama; University of Texas Medical Branch, UNITED STATES

## Abstract

**Background:**

Chikungunya virus (CHIKV) typically causes explosive epidemics of fever, rash and polyarthralgia after its introduction into naïve populations. Since its introduction in Panama in May of 2014, few autochthonous cases have been reported; most of them were found within limited outbreaks in Panama City in 2014 and Puerto Obaldia town, near the Caribbean border with Colombia in 2015. In order to confirm that Panama had few CHIKV cases compared with neighboring countries, we perform an epidemiological analysis of chikungunya cases reported from May 2014 to July 2015. Moreover, to understand this paucity of confirmed CHIKV cases, a vectorial analysis in the counties where these cases were reported was performed.

**Methods:**

Chikungunya cases were identified at medical centers and notified to health authorities. Sera samples were analyzed at Gorgas Memorial Institute for viral RNA and CHIKV-specific antibody detection.

**Results:**

A total of 413 suspected cases of CHIKV infections were reported, with incidence rates of 0.5 and 0.7 per 100,000 inhabitants in 2014 and 2015, respectively. During this period, 38.6% of CHIKV cases were autochthonous with rash and polyarthralgia as predominant symptoms. CHIKV and DENV incidence ratios were 1:306 and 1:34, respectively. A phylogenetic analysis of E1/E2 genomic segment indicates that the outbreak strains belong to the Asian genotype and cluster together with CHIKV isolates from other American countries during the same period. Statistical analysis of the National Vector Control program at the district level shows low and medium vector infestation level for most of the counties with CHIKV cases. This index was lower than for neighboring countries.

**Conclusions:**

Previous training of clinical, laboratory and vector workers allowed a good caption and detection of the chikungunya cases and fast intervention. It is possible that low/medium vector infestation level could explain in part the paucity of chikungunya infections in Panama.

## Author summary

Chikungunya virus (CHIKV) is a mosquito borne pathogen that causes fever with rash and arthralgia, which are often confused with Dengue virus (DENV) infections. It has been reported that when CHIKV colonizes regions without previous circulation, it generally results in explosive human epidemics. In Panama, the first CHIKV infections were detected in May 2014. However, unlike many countries in the Americas, Panama presented with few autochthonous cases during the outbreak. In this study, we investigated the likely reason for the paucity of cases. Low vector infestation level, along with the surveillance programs, preparedness and early outbreak response possibly influenced the low number of cases observed during the Panamanian CHIKV outbreak.

## Introduction

The chikungunya virus (CHIKV, *Alphavirus*, *Togaviridae*) is an RNA single-stranded arthropod-borne pathogen that was first recognized in 1952–1953 in southeast Tanzania and northern Mozambique [1]. In Africa, CHIKV was associated with small outbreaks in rural areas, however, in Asia between 1960–1970, the virus was associated with explosive urban epidemics [2]. Based on the geographical distribution and genetic profiles, CHIKV is divided in three major lineages: the East, Central and South African (ECSA), the West African and the Asian lineages [3]. After a 2004 epidemic in coastal Kenya, CHIKV spread to the Indian Ocean island of La Reunion in 2005–2006, and caused approximately 266,000 cases [4]. In India, a subsequent epidemic reported about 1.4 million cases; several cases were imported to Europe, North and South America [5–8]. A single mutation A226V in E1 gene of the ECSA strains, which increases transmission by *Aedes albopictus*, allowed the emergence of CHIKV Indian Ocean lineage (IOL) [9,10]. The E1-A226V mutation allowed the rapid diversification of CHIKV IOL via a second wave of mutations in the E2; providing evidence that this mutation enhances the likelihood of IOL transmission, and consequently increases the risk of worldwide expansion [11]. Therefore, it was believed that ECSA genotype would be established in the Americas, where *Aedes albopictus* populations are present. However, in 2013 autochthonous CHIKV cases due to the Asian genotype were detected in the French Caribbean Island of Saint Martin. This CHIKV genotype later spread to other Caribbean Islands and the Americas causing epidemics in several countries [12,13].

The introduction of CHIKV into naive populations is followed by an explosive epidemic that affects a large number of people [9,10]. The interaction of some variables most likely favors this phenomenon: 1) susceptible human populations; 2) the presence of both mosquito vectors *Aedes aegypti* and *Aedes albopictus;* 3) and mutations in the virus that increase its infectivity [9,10]. From December 2013 to July 2015, a total of 1,118,763 suspected and 25,463 confirmed autochthonous CHIKV infections were reported in the Americas [14]. In 2015 an atypical presentation of the disease, that included distal extremity necrosis, was reported in Venezuela [15,16].

In Panama, the first imported case of CHIKV was reported in May 2014, and the first autochthonous case in August 2014 [13]. The majority of imported cases were associated with the Colombian, Dominican Republic and Venezuelan epidemics. Two main, but limited outbreaks, were detected in Panama: 1) in Rio Abajo (a county in Panama City) in August 2014 and 2) Puerto Obaldia (a town within an indigenous region near to Colombian border) in January 2015. The incidence rates for 2014 were low (0.5 per 100,000 inhabitants), and no fatalities or severe cases were detected, despite intensive surveillance. This contrasts with the high incidence in 2014 observed in Dominican Republic (5,182.5 per 100,000 inhabitants), Colombia (189 per 100,000 inhabitants) and Venezuela (131 per 100,000 inhabitants) (www.paho.org). This country also reported severe clinical presentations [15,16]. In order to understand these differences and the paucity of CHIKV outbreaks in Panama, we analyzed the CHIKV outbreak response and control in addition to the surveillance data of dengue, chikungunya and *Aedes* vectors between May 2014 and July 2015.

## Methods

### Ethics

All information was obtained during the outbreak response and through the National Dengue and Chikungunya Surveillance programs, thus IRB approval was not necessary to submit (0277/CBI/ICGES/15), however all personal information was removed to perform the analysis and patient identification was codified to respect confidentiality.

### Clinical and epidemiological information

#### Chikungunya case definition

After the detection of the two first imported cases on May 13 and 14 of 2014 in Panama City, Panama [13]. The Ministry of Health (MINSA) implemented an active surveillance of patients with febrile disease that arrived from countries with confirmed CHIKV circulation. The case definition was set up by the MINSA and followed PAHO guidelines and was the following: suspected case: a patient with fever > 38.5°C (101.3°F) and severe arthralgia or acute onset arthritis, which are not explained by other medical conditions [17]. A confirmed CHIKV case was classified as a suspected case with any of the following CHIKV specific tests: viral isolation, detection of viral RNA by reverse transcription-polymerase chain reactions (RT-PCR), detection of IgM in one serum sample (collected during the acute phase or convalescent).

#### Demographics and travel history

The epidemiological notification form included travel history (inside or outside the country) two weeks previous to onset of symptoms. Autochthonous cases were defined as a patient who met the Chikungunya confirmed case definition described above and has not visited epidemic or endemic areas during a two week time period preceding the onset of symptoms.

The clinical, epidemiological and demographic information (day of onset of symptoms, description of symptoms, clinical suspicion of CHIKV or DENV infection, age, sex, district of residence, district of employment) was obtained from the epidemiological notification form and when available from the patients´ clinical records. The spatial and geographical location of each confirmed CHIKV case was represented using ArcGIS software v.10.3.

#### Dengue surveillance

The Dengue Surveillance program was established in 1988 across Panama by MINSA and Gorgas Memorial Institute of Health Studies (ICGES)[18] (S1 Fig). The executive order N° 1617 from 2014 regulates the mandatory notification of the disease to the National Department of Epidemiology (NED) from public and private institutions and the use of World Health Organization (WHO)'s Dengue suspected case definition: a patient with fever and one or more of the following symptoms: headache, retro orbital pain, myalgia, exanthema, rash, vomiting, malaise, leukopenia, jaundice. A confirmed case is a suspected case with a positive dengue test (isolation, RT-PCR, IgM ELISA or secondary IgG ELISA). Since August 2010, the laboratory diagnosis of dengue is decentralized: the health care facilities perform the detection of NS1 antigen and of IgM/IgG antibodies for acute (≤ 4 days of symptoms) and convalescent (≥ 5 days of symptoms) samples, respectively. Quality control tests for the Dengue Laboratories Network from across the country are performed at ICGES with evaluation of around 10% of the monthly positive and negative samples. Acute samples (0–4 days) were also submitted to ICGES for serotype and genotype surveillance (S2 Fig). Acute negative samples are also tested to detect CHIKV viral RNA.

To maintain the quality of the continuous surveillance program for dengue and other arbovirus like chikungunya, yearly, MINSA trained physicians around the country in case definition to detect and report dengue and dengue-like infections. Laboratory training and sample transportation are provided by GMI and MINSA. Finally, the GMI, as the reference laboratory in the country, performs quality control tests through Ibero-American network of emerging viruses (ViroRed), CDC and PAHO for dengue and chikungunya molecular and serological tests.

#### Chikungunya surveillance

The chikungunya laboratory surveillance was centralized to ICGES during the study period. All the acute (≤ 8 days of symptoms onset) and convalescent (≥ 9 days of symptoms onset) suspected chikungunya cases were tested using molecular methods/viral isolation or IgM ELISA (S3 Fig). Every patient that had an acute sample is cited for a second medical visit to obtain convalescent samples. Acute samples should have been sent to ICGES in cold chain within 72h of collection. Usually, acute samples that do not meet these requirements are rejected and not tested. Laboratory results were notified to NED within 72h after receiving the samples (S1 Fig). CHIKV negative samples were also tested to detect DENV infections. In addition, since January 2014, a passive arbovirus surveillance laboratory was started at ICGES. Acute samples with negative results for both DENV and CHIKV, independent of their arrival through the dengue or chikungunya surveillance, were tested using generic primers [19–21], for RT-PCR to detect *flavivirus*, *alphavirus* and *phlebovirus* viral RNA. Any positive sample was sequenced using an Applied Biosystems (Foster City, CA) 410 Genetic Analyzer following the manufacturer’s protocols. Viral sequences were identified using Basic Local Alignment Search Tool (BLAST) Software.

#### DENV and CHIKV infection rates

In order to provide information about differences in magnitude between DENV and CHIKV infections rates, information of all DENV confirmed infections were obtained through 2014 and from January to July of 2015 Dengue national surveillance program, as well as all CHIKV confirmed cases. In brief, incidence rates of CHIKV and DENV infection in 2014 and 2015 were calculated dividing the number of positive cases per 100,000 inhabitants using demographic information of General Comptroller of the Republic of Panama.[22]

### Laboratory assays

#### Molecular testing

Blood samples were received at ICGES, through the DENV or CHIKV surveillance, where serum was separated. RNA was extracted using QIAamp RNA Viral Extraction Kit (Qiagen, Germany) from acute serum samples (≤8 days from onset of symptoms for CHIKV and ≤4 for DENV); samples were tested using real time (RT-PCR) specific protocol for CHIKV (CDC 2009 protocol adapted from Lanciotti, 2007) [7] or DENV[23].

#### CHIKV serologic testing

CHIKV suspected sera samples with ≥ 8 days from onset of symptoms were also tested antibodies against CHIKV IgM using a capture enzyme-linked immunosorbent assay (ELISA), as described previously [24]. Reagents for the ELISA were kindly provided by the Center for Disease Control and Prevention (CDC) vector borne branch at Fort Collins and Pan American Health Organization (PAHO).

#### Viral isolation

Viral isolation was attempted by inoculating 200 μl of acute sera in 12.5 cm^2^ plastic tissue culture flasks of Vero (*Cercopithecus aethiops* kidney normal cells, ATCC® CCL-81™) and C6/36 (*Aedes albopictus* cells, ATCC CRL-1660) were obtained from ATCC. After absorption for 2 hours at 28°C (C6/36) or 37°C (Vero), 5 mL medium with 1% fetal bovine serum was added to each flask, and the cells were incubated at the respective temperatures and 5% CO_2_ and observed daily for evidence of viral cytopathic effect (CPE). Samples were inoculated twice for CPE confirmation. When the CPE was evident, RNA was extracted and tested using Alphavirus genus-specific RT-PCR [19].

### Entomological surveillance and vector control

#### Vector infestation levels

Vector infestation levels were estimated using entomological surveillance of larvae, which consists of a systematic entomological survey by cluster to establish the *Aedes* infestation rates and risk areas of dengue transmission. The surveys are usually performed annually during the months of April, August and December. To analyze all the blocks from a district each survey takes three months on average to complete, and then the next survey begins. The infestation levels are quantified with the survey of 100% of houses in a block and number of containers with larvae per household. Positive houses are reported if at least one recipient has one larva. The larvae Breteau Index (BI) and house index (HI) are usually estimated for the vector infestation level calculation. The BI is estimated with the number of positive containers per 100 houses investigated. While the HI index is estimated by the following equation:

$$HI=\frac{number\;of\;infected\;houses}{number\;of\;inspected\;houses}\;X\;100$$

In this study, the vector infestation level was based on HI, were the risk classification is defined as: High (> 4), moderate (4–2) and low (< 2).

#### Geographical analysis of the vector infestation levels

The vector infestation rates were calculated for each epidemiological week as well as the monthly average from January 2014 to August 2015. The geographic regions analyzed include the districts of Panama and San Miguelito, where the majority of cases were detected. To provide descriptive information about the vector infestation level variation, we performed a box & whisker analysis using the SPSS software package, version 22 (IBM, Corp, New York). A student T-test analysis of mean vector infestation rates was conducted to estimate the differences during May to December 2014 and January to July 2015 (CI, 95%), and differences in abundances of *Ae*. *aegypti* and *Ae*. *albopictus*. Vector control department performed vector identification using ventral brush of the abdomen and subapical spines to distinguish between the larvae of *Aedes aegypti* and *Aedes albopictus*.

#### Vector control measures based on vector infestation levels

In high and moderate risk neighborhoods, the vector control program fumigates all streets every two weeks by car, using Deltametrine (0.27%) and Cyfluthrin (1.5%). For each dengue or chikungunya suspected case in a neighborhood, independent of the vector house index (high, moderate or low risk), the vector control personnel eliminate breeding sites inside houses and peridomiciliary. If some breeding sites can't be eliminated (for example, water supplies for human use), the vector control personnel applies larvicides (Temephos, Diflubenzuron) to these mosquito breeding sites. Direct fumigation and larvicide application inside houses and peridomiciliary is done only for confirmed DENV and CHIKV cases. When more than four cases were reported in a neighborhood, the intervention zone corresponds to the whole district and all inhabitants have to give the permission to the vector control staff to enter the houses. Prevention and control measure information are distributed to the general population using mass media. However, the frequency of mass media message is increased before and during the raining season (May-November) when Dengue cases generally increase.

#### Time-series analysis of the vector infestation rates

In order to determine the general tendency of vector infestation level in the Districts of Panama and San Miguelito during and after CHIKV introduction, we performed a time series analysis. Rates of *Aedes spp* vector infestation were taken as part of the national program for the surveillance of dengue. The data set used in this analysis was collected from January 2014 to July 2015. An exploratory analysis of the data was performed in order to detect missing values, while a descriptive analysis was performed to determine the presence of outliers. Lineal interpolation was necessary in order to reduce the variability produced as a consequence of the outliers and missing data. In order to reduce the variability of the data due to the lack of information during the epidemiological weeks of 14–18, 32–35, 45–53 (2014) and 8 (2015), we performed a linear interpolation and adjusted the cyclical behavior of the time series. Finally, we applied the minimum quadratic method to determine the tendency equation.

### Preparedness and outbreak response

#### Clinical and laboratory training

Following the 2005 outbreak of CHIKV in the Indian Ocean, the CDC and PAHO organized training in 2009 for laboratory diagnosis, which was attended by ICGES staff. More recently, after the first report of autochthonous CHIKV infections in the Americas in December 2013[12], two trainings were organized, one regional training by CDC/PAHO on laboratory diagnosis held at ICGES in June 2014, and another organized by ICGES along with MINSA, and PAHO for national clinical and laboratory personnel. In this training, physicians from Dominican Republic with clinical experience in chikungunya were invited to share experience on case management and laboratory diagnosis in June/July 2014.

#### Surveillance at points of entry

In January 2014, after the CHIKV emergence in the Americas [12], MINSA alerted the airports and terrestrial and maritime ports of entry about CHIKV treat. Additionally, CHIKV case definition, information of countries with confirmed CHIKV epidemics was given. As soon as a febrile case from countries with confirmed CHIKV circulation was identified, medical personnel at the airport notified MINSA, and transferred the patients to medical centers for clinical evaluation and serum sample collection, which was then sent to ICGES.

#### Outbreak response in the neighborhood

After identification of the CHIKV index case in each neighborhood, a comprehensive approach by MINSA was implemented to detect new secondary cases. In Rio Abajo neighborhood, house-by-house surveillance of active febrile cases was performed in the city block where the index autochthonous case lived, and in the surrounding blocks. In parallel, when the medical doctor and ICGES alerted MINSA of a new CHIKV case, the NED notified the Vector Control Department at MINSA, and the house-by-house intervention was performed in less than 48h. Guidance about methods of elimination of mosquito breeding sites was provided to the community of the confirmed cases by distributing educational brochures.

#### Phylogenetic analysis

Amplicons generated from the genus-specific Alphavirus RT-PCRs were purified directly using Qiaquick PCR purification Kit (Qiagen, Germany) and sequenced in both directions with the RT-PCRs primers using an Applied Biosystems 410 Genetic Analyzer (Foster City, CA), following the manufacturer’s protocols. After identification of the viral sequences using Basic Local Alignment Search Tool (BLAST) Software, RT-PCR specific reactions for the E1/E2 gene and primer walking sequencing were performed (Primers sequences for RT-PCR and sequencing were kindly provided by Tiffany Kautz, University of Texas Medical Branch at Galveston).

Consensus sequences covering the structural E1/E2 gene of the 13 CHIKV strains isolated in Panama were aligned with 40 representative homologous sequences from the GenBank library and The European Virus Archive using the MUSCLE algorithm as amino acids and then returned to nucleotides to conserve codon homology [25]. The selection of the best substitution model was based on the Akaike information criterion using the jMODELTEST package [26]. Then a maximum likelihood (ML) tree was then generated using a heuristic search with tree-bisection-reconstruction using the GTR+G+I model [27]. The statistical significance of tree topology was evaluated by bootstrap resampling of the sequences 1000 times. Phylogenetic analyses were undertaken using PAUP* version 4.0,10b [28].

#### Accession numbers

Data are available from the GenBank Nucleotide database at the following accession numbers: KX255061-63 [13] and KX355507, KX355508, KX355509, KX355510, KX355511, KX355512, KX355513, KX355514, KX355515, and KX355516.

## Results

### Clinical and epidemiological description of chikungunya suspected cases

The suspected chikungunya case definition was set up at the beginning of chikungunya outbreak in the Americas to alert all-medical personnel of this new introduction in dengue endemic countries. Overall, 114/413 (27.6%) cases of CHIKV infections were confirmed through CHIKV surveillance. A total of 60.7% were women and 88.6% were more than 15 years old; the mean age was 37.8 years old (SD ± 17.9) (Table 1). All (100%) of patients presented to the health center with fever; the majority presented with polyarthralgia (98%), myalgia (88%), headache (79.3%), chills (77.2%) and rash (76%) (Table 1). A total of 29/413 (7.0%) of the suspected CHIKV cases were confirmed as DENV infections. A high number of suspected cases (65.1%; 269/413) were negative for both viruses.

**Table 1:** Demographic and clinical characteristics of ambulatory cases received for Chikungunya Diagnosis and Surveillance for 2014-2015(Epidemiological week 26)*.

Item	Chikungunya (N = 114)		Dengue (N = 29)		Pathogen not-identified (N = 269)	
	Positive	%	Positive	%	Negative (CHIKV/DENV)	%
Patients						
< 15 years old	13	11.4	5	17.2	41	15.2
Female	63	60.7	14	62.1	147	54.6
Age (years): Mean	37.8	-	34	-	37.9	-
Standard deviation	17.9	-	20	-	19.6	-
Period						
2014	71	62.3	22	75.9	177	65.8
2015	43	37.7	7	24.1	92	34.2
District						
Panama	70	61.4	19	65.5	175	65.1
Comarca/Puerto Obaldia	19	16.7	0	0.0	3	1.1
Arraijan	8	7.0	2	6.9	14	5.2
San Miguelito	5	4.4	1	3.4	20	7.4
Others/No information	12	10.5	7	24.1	57	21.2
Laboratory						
PCR	51	44.7	9	31.0	151	56.1
Meet acute definition^#^	47	92.2	7	77.8	146	96.7
Serology	65	57.0	20	69.0	64	23.8
Meet convalescent definition^#^	36	92.3	12	85.7	43	67.2
Type of case						
Imported	70	61.4	-	-	-	-
Prevalence of symptoms						
Fever	107	100.0	28	100.0	261	98.5
Polyarthralgia	98	98.0	19	79.2	189	87.9
Myalgias	73	88.0	18	78.3	173	84.8
Headache	65	79.3	23	85.2	199	88.1
Chills	61	77.2	19	86.4	185	89.8
Rash	57	76.0	6	28.6	59	42.1
Retroorbital pain	35	33.0	9	32.1	85	32.1
Vomiting/Nauseas	21	38.2	11	47.8	74	46.0
Abdominal pain	9	8.5	4	14.3	30	11.3
Diarrhea	8	7.5	6	21.4	48	18.1
Conjuntivitis	2	4.3	2	11.8	17	13.9

*Total cases 413. The table shows the analysis of 412 cases as one did not have clinical information available.

^#^Samples tested that meet the acute case definition: Chikungunya PCR (≤ 8 days of onset) = 92.1%(47/51), Dengue PCR (≤ 4 days of onset) = 77.8%(7/9), Negative (CHIKV/DENV) PCR = 96.7%(146/151); Samples tested that meet the convalescent case definition: CHIKV IgM Serology (≥ 9 days of onset) = 92.3%(36/39), DENV IgM Serology (≥ 5 days of onset) = 85.7%(12/14); Negative CHIKV and DENV IgM Serology = 67.2%(43/64).

### Epidemiology of chikungunya outbreak in Panama May 2014- July 2015

**Fig 1:**
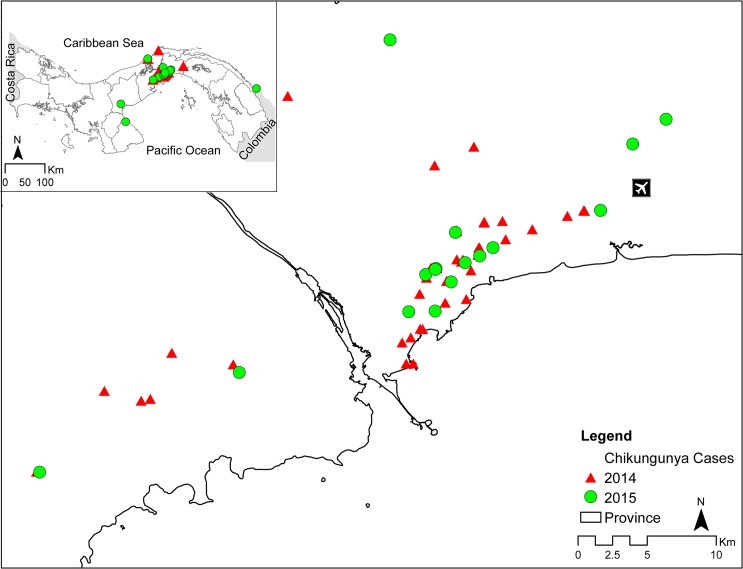
Confirmed chikungunya cases distribution in all Panamanian national territory. Map of Panama with a closer view of Panama City and its surrounding areas. Red triangles represent the cases of 2014 while green dots cases of 2015.

From May (19^th^ epidemiological week) 2014 to July (26^th^ epidemiological week) 2015, a total of 413 patients met the suspected case definition for CHIKV infection in the country. The majority of the suspected CHIKV cases (70.2%, 290/413) were located in the Districts of Panama City and San Miguelito, which are the most densely inhabited districts in the country. Similarly, around 65.8% of confirmed CHIKV infections (75/114) were detected in Panama City and San Miguelito (Fig 1, Table 1).

**Fig 2:**
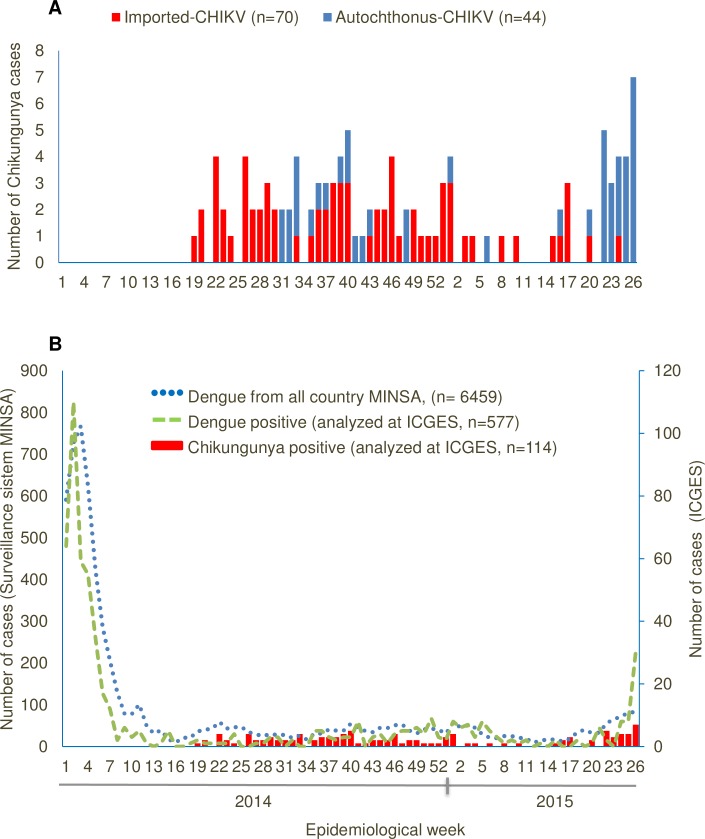
Epidemic curve of chikungunya and dengue cases reported in Panama country from 2014 to July 2015. **A.** Red bar represents Chikungunya imported cases and blue bar autochthonous cases. **B.** Blue dots represent dengue positive cases in the country of Panama from the National Dengue surveillance program (left axis). From this surveillance ICGES detected ~10% (577/6459) of the national dengue positive cases (green broken line, right axis). All the positive Chikungunya cases (red bar, right axis) from the National Chikungunya Surveillance program were analyzed at ICGES.

From all suspected cases, 114/413 (27.6%) were confirmed CHIKV infections, of these 114 cases, 26.3% were detected in 2014 (71/270) and 30.2% in 2015 (43/142) respectively (Table 1). Of these confirmed CHIKV infections, a total of 70/114 (61.4%) were imported infections (Table 1, Fig 2A), 53 of them detected in 2014 and 17 in 2015, respectively.

Two mains CHIKV outbreaks were detected: one in Panama City during 2014 (epi-weeks 31 to 40), with a total of 28 cases, 13 of which were autochthonous and one in 2015 (epi-weeks 22 to 26), which had 19 cases detected in Puerto Obaldia (Figs 1 and 2A). Of the confirmed CHIKV cases, 51 were detected by RT-PCR, showing a concordance of 92.2% with the clinical and molecular diagnosis (≤8 days of symptoms), while 65 were detected by IgM ELISA) with concordance of 92.3% within the clinical and serology diagnosis (≥9 days of symptoms) (Table 1). A total of 29/413 (7.0%), were confirmed cases of DENV infection, and 269/413 (65.1%) were negative for both viruses (Table 1) and for the three mains groups: *alphavirus*, *flavivirus* and *phlebovirus*.

The CHIKV incidence rates were 0.5 and 0.7 per 100,000 inhabitants in 2014 and 2015 (until epidemiological week 26), respectively (Table 2). Data from National Dengue Surveillance system were used to compare CHIKV and DENV infections rates for both years, the ratio indicate that CHIKV and DENV infections were 1:306 and 1:34, respectively. The incidence rates varied between provinces, and Panama province (divided in four health regions: West Panama, Panama, San Miguelito and East Panama) reported the majority of cases (Tables 1 and 2).

**Table 2:** Comparison of the incidence rates of CHIKV and DENV infection in 2014 and 2015 in Panama.

	2014							2015*						
	N		Chikungunya		Dengue		Ratio	N		Chikungunya		Dengue		Ratio
District		Imported	Auchthotonous	Rate^δ^	Cases	Rate			Imported	Auchthotonous	Rate	Cases	Rate	
Panama	1076913	38	16	1.5	1571	145.9	98.2	1098068	14	3	0.3	370	33.7	28.4
Puerto Obaldia	41546	0	0	0.0	0	0.0	-	42395	0	19	44.8	0	0.0	-
Arraijan	262517	7	0	0.0	11	4.2	-	270191	0	1	0.4	6	2.2	36.0
San Miguelito	350949	2	1	0.3	742	211.4	742.0	355429	1	1	0.3	71	20.0	90.0
Others^α^	2181350	6	1	0.05	3193	146.4	3193.0	2209321	2	2	0.1	495	22.4	247.5
Total	3913275	53	18	0.5	5517	141.0	306.5	3975404	17	26	0.7	942	23.7	33.9

*Total cases until epi week 26th

N = Inhabitans, http://www.contraloria.gob.pa/INEC/Publicaciones/Publicaciones.aspx?ID_SUBCATEGORIA=10&ID_PUBLICACION=556&ID_IDIOMA=1&ID_CATEGORIA=3_Accessed October 22th.

^δ^Cases per 100000 inhabitants

^α^Others districts: seven chikungunya cases during 2014 [Colon (1), La Chorrera (1), Chepo (1), Penonome (1) and Missing (2)] and four during 2015 [Colon (2), Los Santos (1) and Nata (1)].

### Chikungunya cases detected through dengue surveillance system

In order to reduce selection bias influence and to increase the possibility of detection of CHIKV cases that were not caught through National Chikungunya Surveillance, samples from Dengue surveillance were also analyzed. All the samples received during the time of the study met the quality control requirement and none was rejected for analysis. From a total of 1489 samples (564 DENV positive and 925 DENV negative) received at ICGES through the National Dengue surveillance system representative from all provinces, we randomly selected and tested a total of 879 dengue negative samples for CHIKV detection (612 from 2014 and 267 until July 2015) (S1 Table). From these, only two samples were CHIKV positive (1/612 in 2014 and 1/267 in 2015).

The national epidemic curve for all dengue positive cases (6459) detected between 2014 to July 2015 in Panama shows an increase in the number of cases at the beginning of 2014, before CHIKV detection in May that same year (Fig 2B). This epidemic curve shows a similar pattern to the epidemic curve of dengue cases detected by ICGES during the same period of time, suggesting that dengue laboratory surveillance at ICGES (8.9% [577/6459] of total cases) represented the behavior through time of all reported dengue cases in Panama. CHIKV infections were detected starting in May 2014 when dengue cases were also lower than the previous months (Fig 2B). Acute DENV and CHIKV co-infection was not detected, however seroconversion for both viruses was detected in 5 patients, and one presented with IgM-dengue and CHIKV detected by RT-PCR[13].

### Vector infestation levels during the chikungunya outbreak

**Fig 3:**
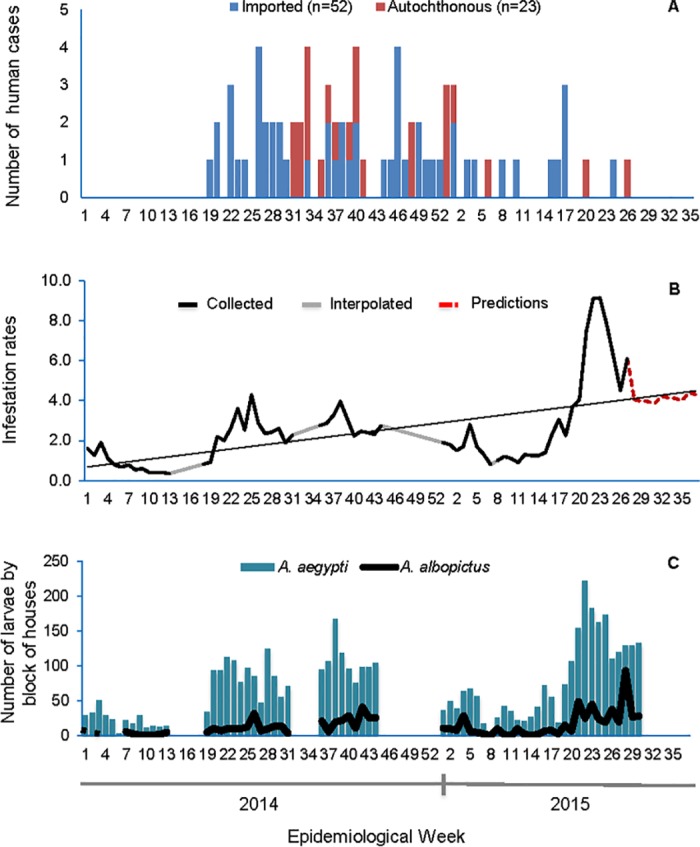
Epidemic curve and vector infestation level reported for each epidemiological week during 2014 and 2015 in Panama and San Miguelito districts. **(A)** Epidemic curve of CHIKV infections (97/107) from epidemiological week 19 in 2014 to 26 in 2015; autochthonous (red) and impor (blue) cases reported. (**B)** Time serial analysis showing data collected (black), interpolated (grey) and predicted (red) of *Ae*. *aegypti* and *Ae*. *albopictus* infestation level. (**C)** Abundance tendency of *Ae*. *aegypti* (blue bars) and *Ae. albopictus* (black line).

To describe vector infestation level of *Ae*. *aegypti* and *Ae*. *albopictus* and their possible relation with the low CHIKV secondary transmission, the vector infestation rates in Panama and San Miguelito were analyzed. Both districts reported most of the Chikungunya confirmed cases (imported and autochthonous), with the majority being detected in 2014 (Fig 3A).

Vector infestation levels observed during the 2014 outbreak fluctuate between 0.4–4.2% (Fig 3B). Low infestation levels were documented mainly during the dry season (January-April) and moderate infestation levels during the rainy season (May-December), where the rate of infection was 4.2%. This corresponds the increase detection of imported CHIKV and secondary transmission (Fig 3A). During 2015, we observed low levels of infestation during the dry season and moderate levels in subsequent months (Fig 3B), when only imported cases were detected (Fig 3A). High infestation levels (8.2 to 14.3%) were observed in the rainy season of 2015. The student t-test applied for 2014–2015 showed no significant difference (P >0.05). When the values of infestation were compared between the months of 2014, it was determined that there was significant difference (P <0.05) between infestation rates only during the months of the rainy season, whereas in the dry season there was no difference. By 2015, only significant difference was observed in the months of April and May due to high incidence and outliers.

The comparison of the standard deviations of the time series with, and without interpolations, corroborated their low variability in the infestation levels through epidemiological weeks (Fig 3B). The minimum quadratic showed that through the eighty-three epidemiological weeks analyzed, the levels of *Aedes sp*. vector infestation increased by a rate of 0.053 per epidemiological week, the graphic show the growing trend behavior observed in the level of infestation (Fig 3B). The abundance analysis shows that *Ae*. *aegypti* is two times more abundant than *Ae*. *albopictus* (P<0.05) (Fig 3C).

### CHIKV infections and vector distribution

**Fig 4:**
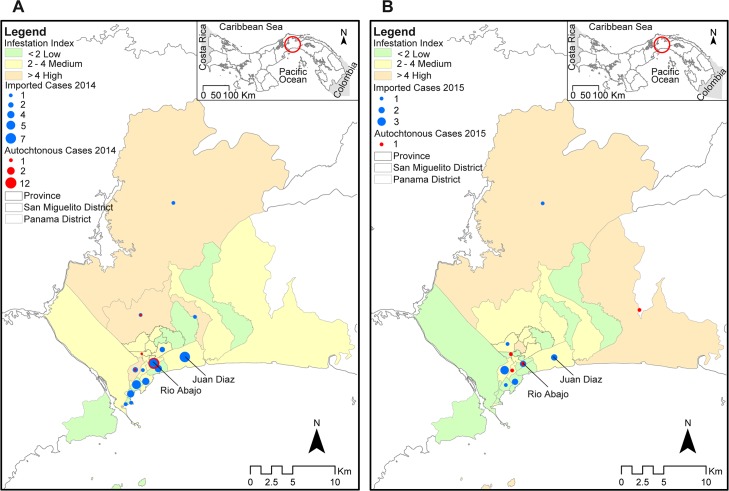
Distribution of chikungunya cases and *Aedes* infestation index in Panama and San Miguelito districts from 2014 and 2015. Maps with imported (blue dots) and autochthonous (red dots) Chikungunya confirmed cases showing the *Aedes* infestation index for each county of Panama and San Miguelito districts (green = low infestation index, < 2; yellow = medium infestation index, 2–4; orange = high infestation index, >4) from May (epidemiological week 19) to December 2014 (**A**) and January to July (epidemiological week 26) 2015 (**B**).

In 2014, imported cases were reported mainly in seven counties in Panama and San Miguelito districts, all of which had low or medium infestation levels, other than the Las Cumbres County, which had a high infestation rate (Fig 4A). Autochthonous cases were detected in only 3 counties: Rio Abajo and Pueblo Nuevo from the District of Panama and Amelia Denis de Icaza from the District of San Miguelito. Rio Abajo County presented a high proportion of imported cases, but low infestation like in Amelia Denis de Icaza County, while Pueblo Nuevo County had medium infestation level. In 2015 (January-July), seven counties had imported cases, all with low or medium infestation levels, other than Las Cumbres. However only four counties reported one autochthonous case for each (Fig 4B), one of them was Rio Abajo again. The infestation rates in Rio Abajo remained between low and medium during 2014 and 2015 respectively (Fig 4B).

To determine if variations over time in vector infestation levels are associated with CHIKV and DENV outbreaks, the CHIKV epidemic curve with imported and autochthonous cases, as well as dengue epidemic curve were superimposed along with the monthly mean infestation rate for Rio Abajo (S4 Fig). The increase of vector infestation levels just before the detection of imported cases was associated with an appearance of autochthonous cases. However, as soon as CHIKV cases were reported, there was a decrease in infestation rates that could be associated with the vector control intervention and educational campaign. Juan Diaz County also maintained a low infestation rate during the period of study. Although imported cases of CHIKV were reported, no autochthonous cases were detected in this County (S3 Fig). Even if vector infestation rates remained medium and low, DENV cases were detected in both counties for most months included in the analysis (S4 Fig).

### Secondary cases detected in the outbreak investigation in Rio Abajo county, Panama City

The imported index case was detected in May 2014 and autochthonous index case was reported in August 15th [13]. In July that year, 3 imported cases and 2 autochthonous cases in the convalescent phase were detected in Rio Abajo County (S4 Fig). From August 9th to 12th, three chikungunya cases during the acute phase were detected, all from the same street in that county. Three convalescent cases were detected also that month. MINSA was notified of laboratory confirmation on September 8th and the active febrile surveillance and mosquito control measures were started on September 11^th^. During this intervention, a total of 15 febrile cases were detected (onset of symptoms ranged from 0 to 8 days), all negative for dengue and two of them were confirmed as CHIKV infection. In total from August 15th to October 6th 2014, 37 acute cases from Rio Abajo were tested for CHIKV infection, of which 7 were confirmed by RT-PCR.

### Phylogenetic analysis of chikungunya viral strains

**Fig 5:**
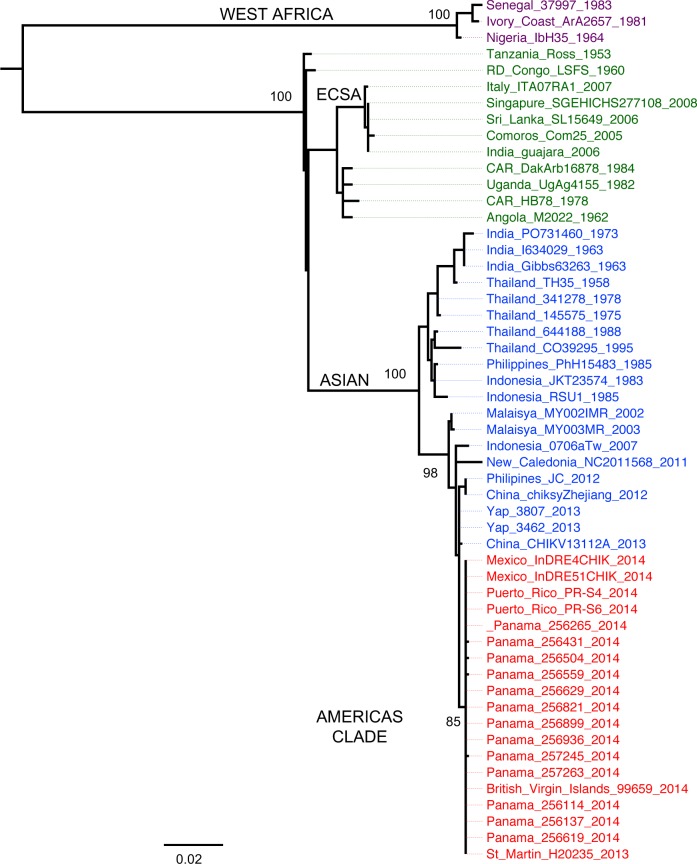
CHIKV phylogenetic tree. Maximum Likelihood (ML) tree of 53 CHIKV sequences based in the E1/E2 protein gene fragment of 995 nucleotides. The three major viral lineages and the American Clade are shown. British Virgin Island's and strains detected in Panama (imported and autochthonous) are in red like all the American clade strains. Virus labels include country of isolation, strain designation, and year of isolation. The evolutionary relationships were undertaken with the GTR+G+I model. Bootstrap values are shown in major branches. All horizontal branch lengths are drawn to scale (bar = 0.02 nucleotide substitutions per site). The tree is midpoint rooted for purposes of clarity only.

The optimal Maximum-likelihood (ML) tree, based on a 975 nt segment of the E1/E2 genes sequences of 53 CHIKV strains (Fig 5), shows that the 13 Panamanian imported and two autochthonous isolates (Genbank accession number KX255061-63 [13], KX355507-16) cluster together within the American clade of the Asian lineage. The Panamanian strains (256821, 257245 and 257263) were obtained from febrile patients that came from Venezuela, while the strain 256899 was obtained from a patient traveling from El Salvador. The majority of imported strains included in our study were obtained from patients with febrile disease that came from Dominican Republic. Two strains were autochthonous to Panama (256619, 256629). Sequences from all Panamanians strains (imported and autochthonous) included in our study are nearly identical to each other, and cluster together with strains isolated in Saint Martin in 2013 [12] and the British Virgin Islands during 2014 [29]. No known mutations of vector adaptation were found in the analyzed E1/E2 sequence.

## Discussion

While many tropical Caribbean and Latin American countries that reported autochthonous infections after 2013 have experienced explosive CHIKV epidemics [30], in Panama, from May 2014 to July 2015, we confirmed only 46 autochthonous CHIKV cases (44 through CHIKV surveillance and 2 through dengue surveillance), that corresponds to the 38.6% of total CHIKV confirmed cases (Table 1 and S1 Table). Clinical and laboratory findings of CHIKV infections in the Panamanian outbreak were similar to those reported in previous epidemics [31–33]. The most predominant signs and symptoms observed in CHIKV Panamanian cases were rash and polyarthralgia, similar to previous reports [31–33]. In previous reports polyarthralgia was more prevalent in CHIKV when compared to DENV infections [34]. In the Panamanian cases, no fatalities or atypical clinical presentations, such as extremity necrosis occurred [15,16]. Therefore, suspected chikungunya cases could be misdiagnosed here as suspected dengue cases. Even though the viremia is higher and lasts longer than dengue, our data show that even through both surveillance programs, few cases of CHIKV were detected [35].

High CHIKV incidence rates and severe cases were reported in several countries of Latin America from July 2014 to July 2015: Dominican Republic: 5,182.5 and 0.6 per 100,000 inhabitants; El Salvador: 2,135.4 and 375.8 per 100,000; Guatemala: 178.1 and 50.8 per 100,000, Honduras: 66 and 607.1 per 100,000, Nicaragua: 70.6 and 331.9 per 100,000, Colombia: 189 and 612 per 100,000, and Venezuela: 131 and 42 per 100,000, respectively. The overall mortality rates in Latin America was 194 in 2014 and 41 in July 2015 [14]. In comparison, in Panama, the incidence rates were 0.5 and 0.7 per 100,000 inhabitants in 2014 and 2015, respectively.

In both years 2014 and 2015, the CHIKV incidence rate was lower compared to DENV. The marked difference observed between CHIKV and DENV in 2014 is probably due to the high number of dengue cases detected during January-April 2014 that corresponded with a dengue outbreak that began just before Chikungunya detection (Table 2). Dengue is an endemic disease in Panama, and about 23 years have passed since reintroduction. This fact may influence physicians’ criteria for differential diagnoses. The sensitivity of dengue diagnosis through the decentralized surveillance should be greater than the recent implemented centralized CHIKV surveillance. In addition, the majority of DENV infections are asymptomatic and sustained DENV transmission by people who are infected without developing detectable clinical symptoms have been proposed [36]. This fact may reduce the effectiveness of traditional control methods that in contrast could be effective in the control of CHIKV infections as the majority of cases are symptomatic [2,31]. Future studies need to be conducted to determine if there are ecological or virus-specific constraints on DENV or CHIKV, due to usage of the same vectors and reservoir.

The majority of CHIKV cases were found in the Panama City metropolitan area (Districts of Panama and San Miguelito). This is likely related to the fact that the most densely populated areas are Panama City and San Miguelito, and both have a high proportion of immigrants inhabiting or working there. The majority of imported cases in 2014 were from Dominican Republic and in 2015 from Colombia and Venezuela. The latter two countries had reported CHIKV outbreaks during that year [14]. Imported CHIKV infections were not reported in Puerto Obaldía during 2015. Human migration of native communities through the Panamanian-Colombian border is very usual. This migration is most likely not reported; therefore, it is difficult to determine if these cases were autochthonous. As Colombia had an important CHIKV epidemic during 2015, it is possible that migration over this border was the most likely way the virus was introduced in the east side of the country.

In this study, we sought to provide information about viral genetic differences that could explain the paucity of CHIKV cases observed in Panama. However, our phylogenetic analysis indicates that these CHIKV strains from the Asian genotype are nearly identical to those strains that are circulating in the rest of the Americas [13,29]. These results suggest that a single introduction of CHIKV from the Asian genotype in the Caribbean islands in 2013 spread to the rest of the Americas, and that Panama is not an exception.

No mutations of vector adaptation in the E1/E2 genes of CHIKV strains included in our studies were found. Further studies of 3’UTR of Panamanian CHIKV strains, should be addressed because it has been proposed that this genomic region may reduce the fitness of the Asian genotype for efficient transmission by mosquitoes [37]. Together, this information suggests that the naive population, along with the presence of *Aedes aegypti* [38] were the major forces that facilitate the dissemination of the Asian genotype through the Americas and not a specific mosquito adaptation like in the La Reunion outbreak [10].

As no specific adaptive mutations were found, other possible explanations for the paucity of Panamanian CHIKV cases are: a) heterologous alphavirus antibodies cross-protect against CHIKV infection and/or disease; b) low level of vector infestation before and after CHIKV introduction; c) and the early case detection and implementation of control measures.

Experimental infections in mice with Mayaro virus (MAYV) and the Alphavirus encephalitis viruses (VEEV and EEEV) have shown cross-protection that seems to last around 2 months [39]. Cross-reactivity between MAYV and CHIKV has been reported [40]. However, it is not clear whether previous infections with VEEV, MAYV or Una virus (UNV) have an impact in CHIKV infection or disease. Moreover the distribution of the main vectors of VEEV, EEEV and probably MAYV and UNV is sylvatic and rural [41,42]. This contrast with the current distribution of *Ae*. *albopictus* and *Ae*. *aegypti* in Panama that are mainly urban and peri-urban [41] and with the distribution of the CHIKV confirmed cases. Further studies should explore the effect of Alphavirus cross-protective immunity in the pattern of CHIKV emergence observed in Panama.

Experimental studies have shown that *Ae*. *aegypti* and *Ae*. *albopictus* efficiently transmit CHIKV of the Asian genotype with a major fitness in *Ae*. *aegypti* [43]. Our analysis of vector infestation levels shows a predominant low to moderate risk for CHIKV epidemics. The detection of only 3 secondary cases during the main CHIKV outbreak in Panama City (Rio Abajo County) suggests that the observed infestation levels along with interventions after a suspected case detection may play a role in the observed pattern of CHIKV outbreak in Panama. Nevertheless, we were not able to test the hypothesis that rapid vector control measures impacted the dynamics of CHIKV transmission in Panama; little information of vector infestation levels in specific locations before and after the control response was available. However, biochemical studies have shown that strains of *Ae*. *aegypti* in Panama City are sensitive to the insecticides used currently for control campaign [44]. The sensitivity of the more abundant Panamanian vector to insecticides and the low number of CHIKV cases in the community following vector control measures suggest that these local control measures applied by MINSA in areas close to confirmed cases were efficacious to limit the number of human cases and the expansion of CHIKV throughout the country. Additionally, because behavior and lifestyle have been proposed to increase or restrict the transmission of DENV rather than the effects of climate in some places [45], the role of lifestyle in the distribution pattern of CHIKV infections observed in Panama, should be addressed in future studies.

The population of *Ae*. *aegypti* appears to be two times more abundant than *Ae*. *albopictus* in Panama City where the majority of cases occurred. *Ae*. *albopictus* has expanded across Panama and predictions indicate that it will colonize the entire Pan-American highway, potentially increasing the area of CHIKV transmission [41]. However the experimental and field studies of vector competence and transmission of Asian CHIKV strains [38,43] suggest that *Ae*. *aegypti* may be currently the principal vector of CHIKV transmission in Panama. Variations in the temperature have shown to reduce *Ae*. *aegypti* vector competence for DENV transmission [46], further studies should address the effect of temperature and humidity in CHIKV transmission, a factor that could add to the small number of CHIKV infections observed in Panama as CHIKV emerged in Panama during the drought in El Niño season.

The early recognition of CHIKV with the subsequent measures could have limited the autochthonous cases in Panama. The first imported cases were detected during the viremic phase, with one case detected at the airport [13]. The available epidemiological data shows that around half of CHIKV cases in Panama were detected during the first days of symptoms. This suggests that the surveillance program was able to detect clinical chikungunya cases during the acute phase. DENV and CHIKV laboratory surveillance plays an important role in Panama. All suspected chikungunya cases were laboratory tested, whereas around 50% of Dengue cases are laboratory confirmed (MINSA-NED.). This differs from the situation in other countries during 2014, like El Salvador or Colombia that had only 157 confirmed cases from the 135,226 CHIKV suspected cases, or 611 confirmed from 90,481 suspected respectively [14]. Moreover, ICGES not only tested all chikungunya suspected cases, in addition its sensibility to detect viral RNA in acute samples was increased by using specific CHIKV detecting methods complemented with genus-specific alphavirus RT-PCR in dengue suspected cases that were dengue negative. The epidemiological surveillance algorithm establishes testing of convalescent paired samples to confirm positive and negative CHIKV results, to avoid false negatives results by RT-PCR due to an inaccurate onset symptoms description, low viral load or sample handling. Although patients were encouraged to attend a second medical visit to obtain the convalescent sample, few participated, especially as symptoms disappeared. For the convalescent paired samples obtained, serological tests were performed as a confirmation of acute samples. PRNT was suggested by PAHO for diagnosis confirmation and would be helpful to confirm seroconversion, especially when circulation of chikungunya has to be proven in a new country[17], this technique was not used at the beginning of the outbreak in Panama as Chikungunya first detected cases were acute, thus Chikungunya circulation was proven in Panama by molecular methods and viral isolation[13]. Once Chikungunya circulation was confirmed in Panama, the CHIKV surveillance program was adapted to the capacity of GMI laboratory and Chikungunya PRNT was not available to confirm serologic results at the time of the outbreak. However, this limitation in the chikungunya surveillance was also shared by most Latin American countries.

Vector control responses at the community level began before the laboratory confirmation, with the notification of a suspected case by the physician to MINSA. After case confirmation and notification, the vector control personnel applied intradomiciliary fumigation in order to complete the response. The algorithm set for the notification of confirmed cases includes notification (around 24 to 72 h) from the laboratory to the treating physician as well as at the local level where the cases are reported and at the National Epidemiology Department at MINSA that informs the Vector Control Department, which in less than 48h organized a complete vector control response with implementation of effective control measures, such an isolation and mosquito control in domiciliary, peridomiciliary and the surrounding community [47]. It is possible that the low number of CHIKV cases in Panama was influenced by the early response after detection of each new case [48]. Quality controls of vector control measures were done every six months. During each evaluation, the calibration of the spraying equipment, calibration of the diameter of the mouthpiece and the drop size used in fumigation was done, as well as control of the security of equipment and chemical usage. Human resource was also retrained every year. Population acceptance of fumigation was high as there are familiar with this procedure since Panama sanitation measures during Panama Canal construction [49], however there are always some closed houses that can not be analyzed intradomiciliary. Vector resistance pilots were done every year by GMI entomologists.

Our study has several limitations. First, the chikungunya suspected case definition was based in fever and arthralgia or arthritis in order to differentiate chikungunya from dengue infections, this may represent a selection bias, as the symptoms may be variable in some chikungunya infections [50]. However, samples from patients with dengue-like disease were also tested even if the physician did not request chikungunya test. Underreporting of cases is also possible, even if notification is mandatory; this is not a total guarantee that all suspected cases were reported. However, all medical institutions in Panama have epidemiologists that follow notification of cases daily and increase the surveillance during outbreak response. A small number of samples from remote areas were received at ICGES during the study. This is possibly due to the long distance and logistical difficulties that arise for transport of samples. DENV surveillance is able to detect an increase of cases in remotes areas and transport samples to ICGES during an outbreak response, however an increase of cases was not reported during our study. Most cases were treated as ambulatory cases in primary care centers where the majority of information was obtained from the epidemiological notification form. Little information on the clinical evolution is currently available, and the rate of chronic CHIKV infections in Panama is unknown, as most patients did not return to the health facility for follow-up after their first visit. This information contrasted with the high proportion of patients with sequelae that were reported in hospitals from countries like Dominican Republic and El Salvador [51]. Similarly, in this study only the clinical symptoms described in the notification form were reported, and not chemical or hematologic test were performed, in consequence correlation between clinical presentation, thrombocytopenia or leucopenia was not possible. The Panamanian vector surveillance during the outbreak was performed using Breteau index that is a larval based index, this index have been found to be inadequate as indicators for dengue virus transmission [52–54]. Indeed, a study have been proposed that adult mosquitoes index correlate better with positive dengue cases in Iquitos, Peru [53], however no study has been done to correlate these differences indices in Panama with dengue or chikungunya cases and larvae index have also shown to correlate with dengue epidemics in Cuba [55]. This discrepancy in mosquito estimator to predict outbreak, suggest variations of correlation among countries. MINSA are considering the use of pupa index instead of larvae; nevertheless, this has not yet been implemented. Finally, detection of the circulating virus in mosquitos was not performed during the response; in consequence, there is no estimate on the rates of infected mosquitos for DENV and CHIKV that could help define risk areas of transmission.

In summary, our data suggest that Panama presented a small and limited outbreak compared with others Latin American countries. Panama was able to maintain low to medium vector infestation levels in the majority of the counties or communities with imported CHIKV cases, and this, along with the interventions after identification of CHIKV infections, as well as other epidemiological conditions, could play a role in the low numbers of cases observed. The constant possibility of new imported CHIKV cases and the introduction of Zika virus in 2015 [56] will continue to be a threat for the surveillance and vector control programs. The capacity to maintain the observed pattern will depend on the preservation of low to medium infestation levels and the sustainability of the early case detection system of dengue-like arboviral diseases, as well as the subsequent implementation of vector control measures after detection of new cases before bigger epidemics.

## Supporting information

S1 Fig Flow chart for the notification of chikungunya or dengue cases of the Ministry of Health for Panama. (TIF)

S2 Fig Flow chart of National Dengue Surveillance Laboratory Program. (TIF)

S3 Fig Flow chart of National Chikungunya Surveillance Laboratory Program. (TIF)

S4 Fig Epidemic curve of chikungunya and dengue cases and vector infestation rates reported in Rio Abajo and Juan Diaz counties from 2014 to July 2015. **A.** Rio Abajo County **B.** Juan Diaz County. Red bar represents chikungunya imported cases and green bar autochthonous cases. Grey bar represent dengue cases and line blue represent the infestation index mosquitos (percentage).
(TIF)

S1 Table Dengue and chikungunya detection in samples received by National Dengue Surveillance Program during 2014 until July 2015 (Epidemiological week 26). (DOCX)
