# Supplementary figures and images for: Unusual pattern of chikungunya virus epidemic in the Americas, the Panamanian experience
Source: PLoS Negl Trop Dis. 2017 Feb 21;11(2):e0005338. doi: 10.1371/journal.pntd.0005338 (PMC5336303; doi:10.1371/journal.pntd.0005338)

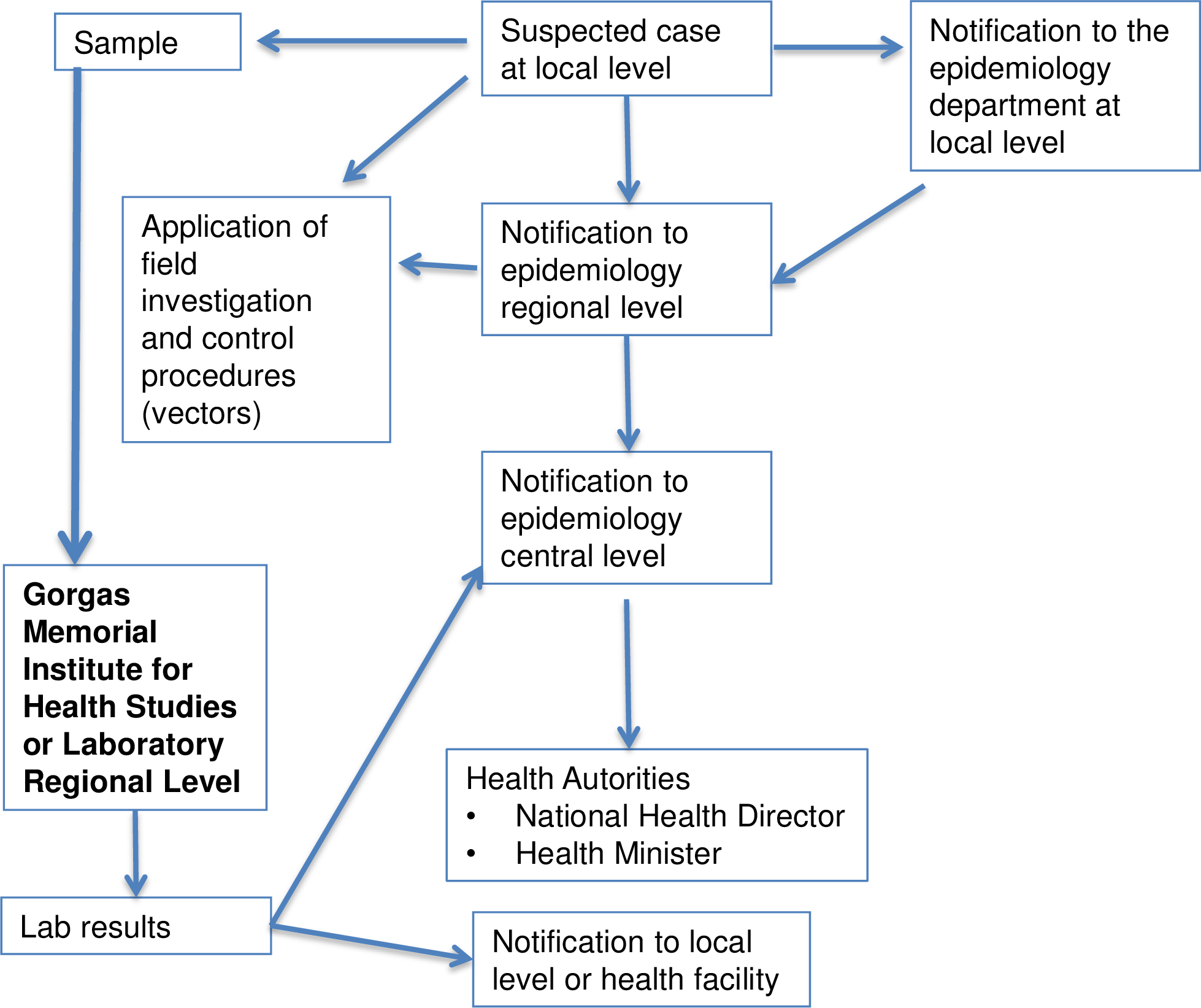

Supplement: S1 Fig — (TIF) [file pntd.0005338.s001.tif]

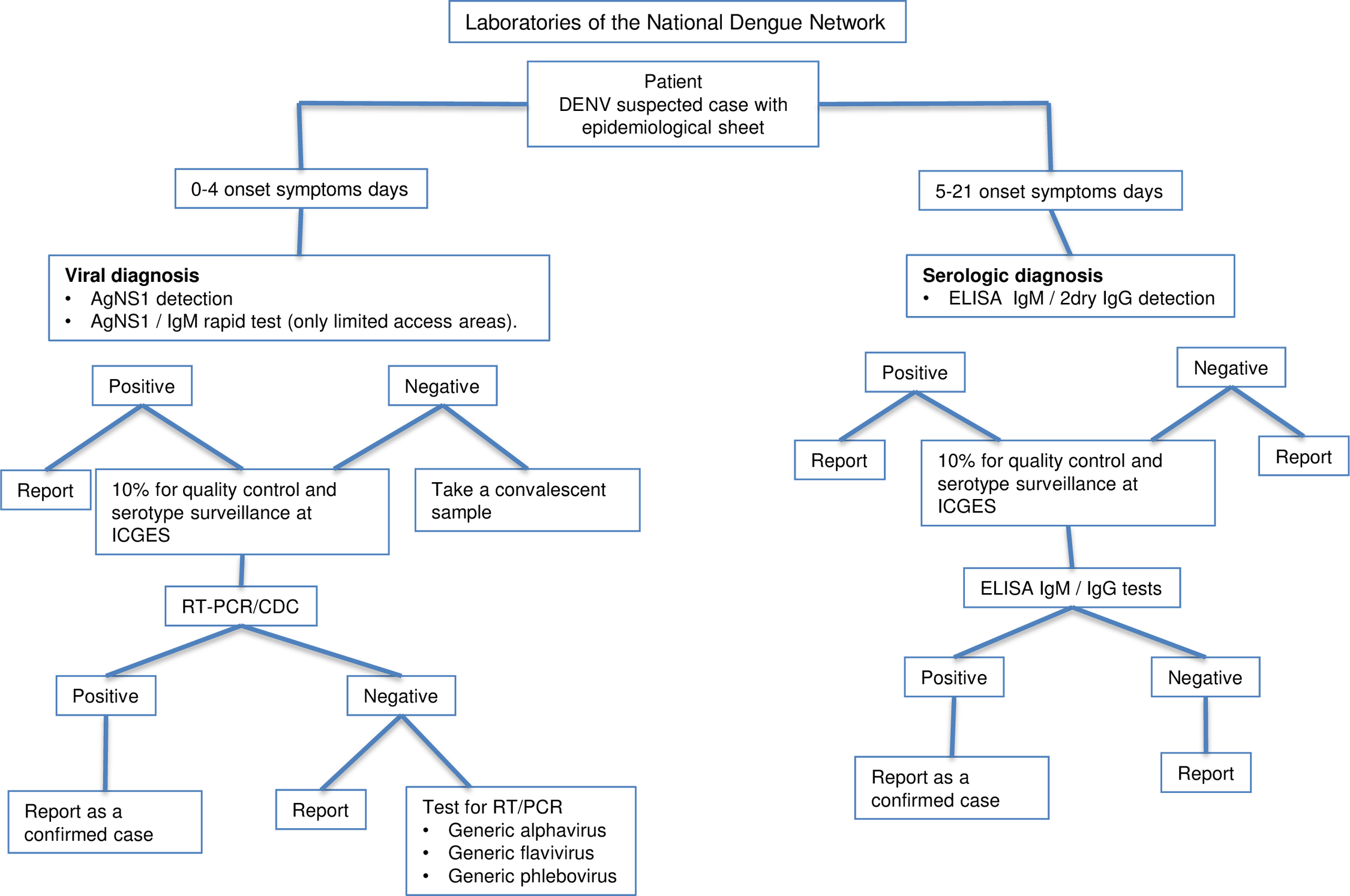

Supplement: S2 Fig — (TIF) [file pntd.0005338.s002.tif]

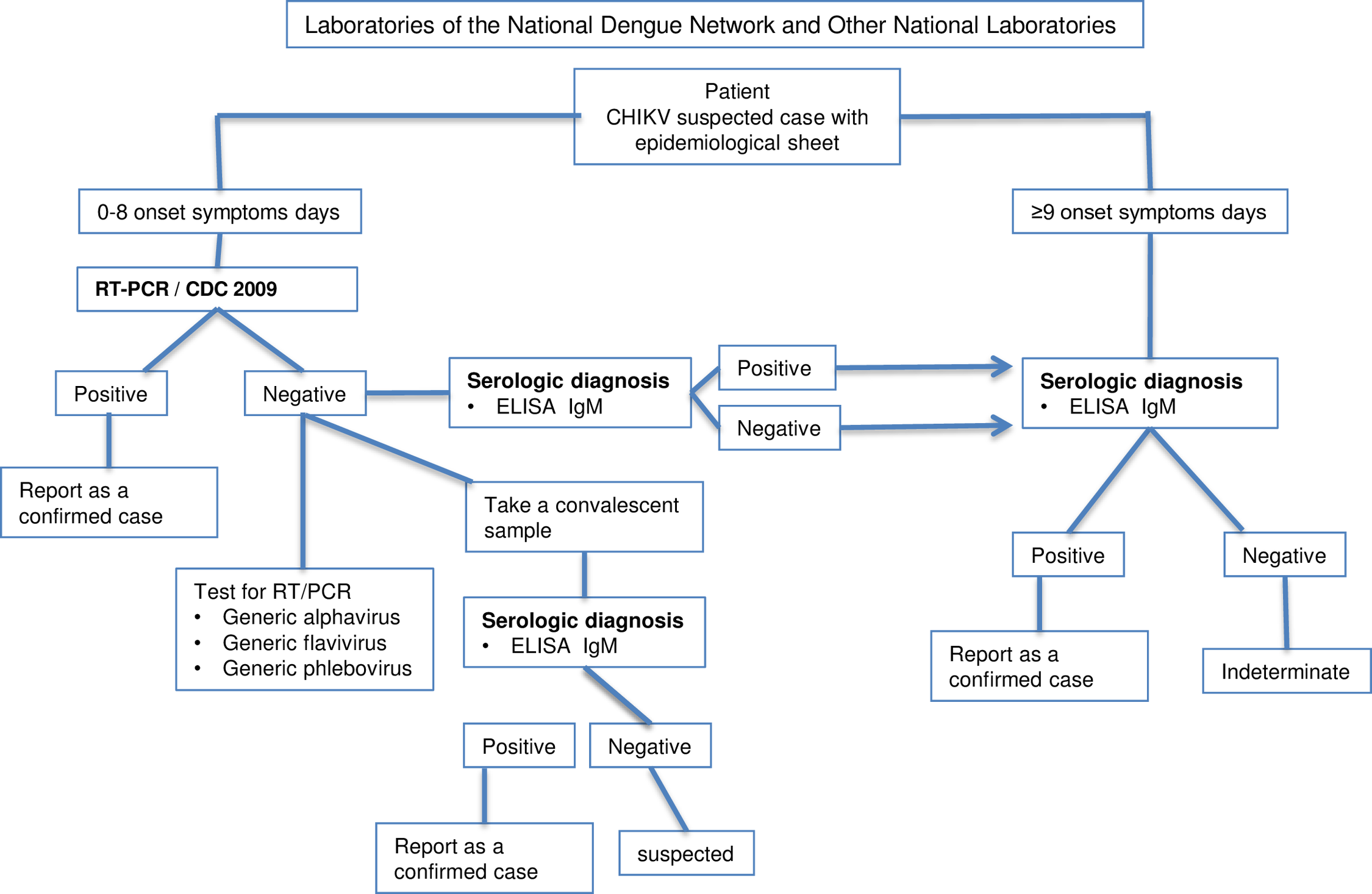

Supplement: S3 Fig — (TIF) [file pntd.0005338.s003.tif]

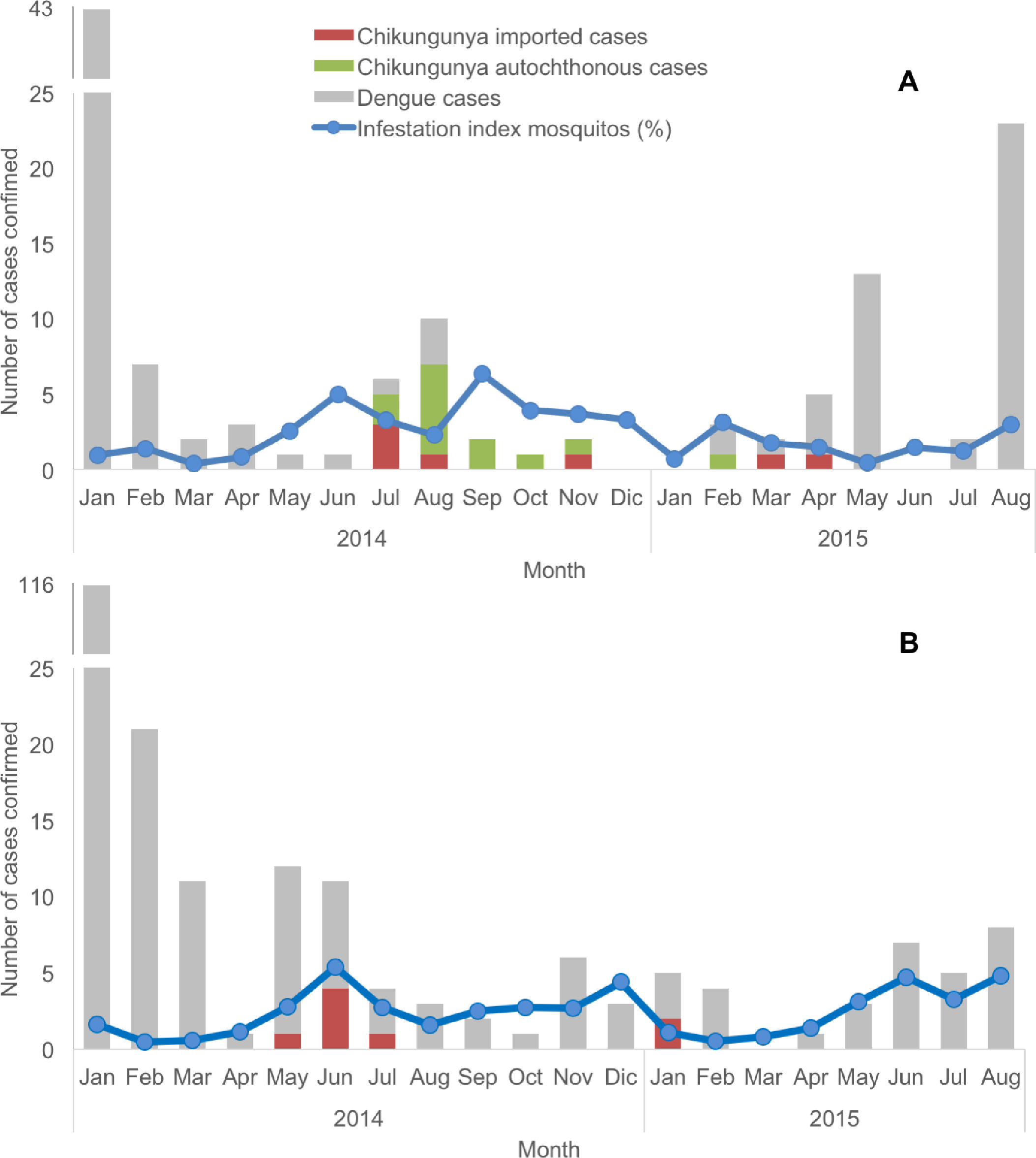

Supplement: S4 Fig — A. Rio Abajo County B. Juan Diaz County. Red bar represents chikungunya imported cases and green bar autochthonous cases. Grey bar represent dengue cases and line blue represent the infestation index mosquitos (percentage). (TIF) [file pntd.0005338.s004.tif]
