# Supplementary material for: Unusual pattern of chikungunya virus epidemic in the Americas, the Panamanian experience
Source: PLoS Negl Trop Dis. 2017 Feb 21;11(2):e0005338. doi: 10.1371/journal.pntd.0005338 (PMC5336303; doi:10.1371/journal.pntd.0005338)
Supplement: S1 Table — (DOCX) [file pntd.0005338.s005.docx]

| **S1 Table 1.** Dengue and chikungunya detection in samples received by National Dengue Surveillance Program during 2014 until July 2015 (Epidemiological week 26) | | | | | | | | | | | | | | | | |
| --- | --- | --- | --- | --- | --- | --- | --- | --- | --- | --- | --- | --- | --- | --- | --- | --- |
|  | Dengue | | | | | | | |  | Chikungunya | | | | | | |
|  | Total* | 2014 | |  |  | 2015 | | |  | 2014 | |  | 2015 | | |  |
| District |  | Positive | n | % |  | Positive | n | % |  | Positive | n | % | Positive | n | % |  |
| Bocas del Toro | 16 | 7 | 16 | 43.8 |  | 0 | 0 |  |  | 0 | 7 | 0.0 | 0 | 0 |  |  |
| Chiriqui | 11 | 1 | 8 | 12.5 |  | 1 | 3 | 33.3 |  | 0 | 5 | 0.0 | 0 | 1 |  |  |
| Cocle | 150 | 79 | 125 | 63.2 |  | 7 | 25 | 28.0 |  | 0 | 46 | 0.0 | 0 | 18 | 0.0 |  |
| Colon | 29 | 1 | 21 | 4.8 |  | 0 | 8 | 0.0 |  | 0 | 19 | 0.0 | 0 | 7 | 0.0 |  |
| Darien | 13 | 2 | 9 | 22.2 |  | 2 | 4 | 50.0 |  | 0 | 7 | 0.0 | 0 | 2 |  |  |
| Herrera | 259 | 123 | 222 | 55.4 |  | 18 | 37 | 48.6 |  | 0 | 99 | 0.0 | 0 | 18 | 0.0 |  |
| Kuna Yala | 20 | 1 | 2 | 50.0 |  | 5 | 18 | 27.8 |  | 0 | 1 |  | 0 | 9 | 0.0 |  |
| Los Santos | 49 | 22 | 36 | 61.1 |  | 7 | 13 | 53.8 |  | 0 | 13 | 0.0 | 0 | 6 | 0.0 |  |
| Panama Este | 76 | 2 | 28 | 7.1 |  | 35 | 48 | 72.9 |  | 0 | 21 | 0.0 | 0 | 12 |  |  |
| Panama Metropolitana | 594 | 131 | 408 | 32.1 |  | 32 | 186 | 17.2 |  | 1 | 270 | 0.4 | 1 | 139 | 0.7 |  |
| Panama Oeste | 136 | 65 | 117 | 55.6 |  | 1 | 19 | 5.3 |  | 0 | 50 | 0.0 | 0 | 16 | 0.0 |  |
| San Miguelito | 96 | 16 | 68 | 23.5 |  | 0 | 28 |  |  | 0 | 52 | 0.0 | 0 | 28 |  |  |
| Veraguas | 15 | 1 | 13 | 7.7 |  | 0 | 2 | 0.0 |  | 0 | 11 |  | 0 | 2 | 0.0 |  |
| Reservation^Ω^ | 25 | 5 | 16 | 31.3 |  | 0 | 9 |  |  |  | 11 |  |  | 9 |  |  |
| Total | 1489 | 456 | 1089 | 41.9 |  | 108 | 400 | 27.0 |  | 1 | 612 | 0.2 | 1 | 267 | 0.4 |  |
| *Total received samples during 2014-2015(Epidemiological week 26) | | | | | | | | | | | | | | | |  |
| ^Ω^Reservation= Other indigenous regions (Ngöbe-Buglé and Embera-Wounaan) and missing. | | | | | | | | | | | | | | | |  |
